# Examining the Nutrition, Oral Mucositis, and Gastrointestinal System Symptoms of Intensive Care Units Patients Receiving Enteral and Parenteral Nutrition

**DOI:** 10.5152/tjg.2023.22125

**Published:** 2023-08-01

**Authors:** Kevser Sevgi Ünal Aslan

**Affiliations:** 1 Department of Fundamentals of Nursing, Osmaniye Korkut Ata University Faculty of Health Sciences, Osmaniye, Turkey

**Keywords:** Enteral nutrition, oral mucositis, parenteral nutrition, nursing care, intensive care

## Abstract

**Background/Aims:**

This descriptive study aims to examine the complications that might develop in patients who receive enteral or parenteral nutrition treatment in intensive care unit in this process and to examine the nutritional status, oral mucositis, and gastrointestinal system symptoms among patients who receive enteral or parenteral nutrition treatment in intensive care unit.

**Materials and Methods:**

A sample of this study consists of 104 patients who received enteral or parenteral nutrition treatment in intensive care units between January and June 2019. The data were collected face to face by using Sociodemographic Form, constipation severity scale, Mini Nutritional Assessment Scale, Mucositis Assessment Scale, visual analog scale, and gastrointestinal system Symptoms Scale. The results were calculated as numbers, percentage, SD, and mean values.

**Results:**

Among the participating patients, 67.4% were older than 65 years, 55.8% were female, 42.3% were receiving treatment in internal medicine intensive care units, and 43.4% had severe mucositis. It was determined that 31.7% of the patients receiving treatment in intensive care units required nutrition treatment. It was determined that patients receiving parenteral nutrition had more symptoms such as gastrointestinal system symptoms, mucositis, constipation, and colonic inertia.

**Conclusions:**

It was determined that when compared to the patients receiving enteral nutrition, the patients receiving parenteral nutrition had higher scores in mucositis, visual analog scale pain, Mini Nutritional Assessment Test, constipation, obstructive defecation, colonic inertia, and gastrointestinal symptom total scores.

**Main Points**

- A statistically significant difference was found in terms of Mini Nutritional Assessment Test total score according to the enteral and parenteral nutrition status of the patients treated in the intensive care unit (*Z* = –6.611; *P* = .000).
- A statistically significant difference was found in terms of gastrointestinal system total scores according to enteral and parenteral nutrition status (*Z* = –4.910; *P* = .000).
- The gastrointestinal symptom total scores of the parenteral feds were statistically significantly higher than the enteral feds.
- It was determined that the gastrointestinal symptom severity of parenteral feds was higher than enteral feds.

## INTRODUCTION

For the patients receiving treatment in intensive care units, nutrition support mainly aims to prevent the malnutrition. Malnutrition can cause problems in the immune system, respiration, and functional status, as well as higher caring costs because of the longer time in ventilation support.^1^ Enteral nutrition (EN) and parenteral nutrition (PN) are used in order to eliminate the malnutrition for the patients, who cannot orally feed. Parenteral nutrition is a nutrition support method that is preferred in cases that EN is contraindicated or cannot be tolerated.^2-4^

Parenteral nutrition is considered complementary to EN and, thus, these 2 methods are not alternative but complementary to each other.^5,6^ Since the clinical appearances of intensive care patients are very heterogeneous, their nutritional statuses may also vary depending on their situations. In a study carried out on intensive care unit patients having high malnutrition risk, it was determined that the intake of a minimum of 800 kcal reduced the mortality rates. Moreover, in a study carried out on patients after gastrointestinal system (GIS) surgery, it was determined that the patients having malnutrition risk had better survival rates when receiving sufficient calorie or protein support via EN or PN.^7^ For this reason, it is very important to select the appropriate nutrition method and to be aware of the potential effects of the selected method on the patient. It is important for all nurses to follow the evidence-based procedures during their daily practice regarding enteral and PN and to use them in the treatments.

## MATERIALS AND METHODS

### Trial Design

This descriptive study was carried out in order to examine the nutrition, oral mucositis, and GIS of intensive care patients receiving enteral or PN support.

### Study Settings

This study was carried out in the intensive care unit of a hospital between January and June 2019.

### Sample Size

The universe of this study consisted of patients receiving treatment in intensive care unit between January and June 2019. The total number of patients receiving treatment in intensive care units between these dates is 180. In this study, no sampling process was conducted, but 109 patients (*n* = 109) meeting the inclusion criteria. Moreover, 5 patients, who were transited from enteral or PN to oral feeding or had GIS bleeding or diarrhea within 1 week of monitoring period, were excluded. Therefore, the study was completed with 104 patients.

The postpower analysis of this study was performed using G Power Version 3.1. Effect size was calculated to be *f* = 0.71. The sample size of 104 patients was found to be within the sufficiency limits for *α* = 0.05 and 1 – *β* = 0.99 (power of 95%).

### Participants

The inclusion criteria of the patients were determined as patients aged 18 and over, receiving treatment in the hospital’s intensive care units (Surgery, Internal Medicine, CVS Intensive Care Unit) for at least 3 days, receiving EN or PN.

Exclusion criteria from the study were those with mechanical obstructive defecation, paralytic ileus, generalized peritonitis, acute pancreatitis, inflammatory bowel disease, gastrointestinal bleeding, diagnosis and jejunostomy of gastric cancer and esophageal cancer, impaired mental functions to prevent communication, hearing problems, and those who do not speak Turkish. Patients’ nutrition both enterally and parenterally was not included in the study.

In addition, the EN patient group consisted of patient’s nutrition with percutaneous endoscopic gastrostomy (PEG) and nasogastric tube (NG). Also, parenteral nutrition of patients consisted of peripheral venous nutrition and central venous nutrition. Combination nutrition (parenteral + enteral) patients were not included in the study.

### Instruments

The data collection was performed using Introductory Information Form, Mini Nutritional Assessment Test (MNA), visual analog scale (VAS), RMI, CSS, and GIS scale.

Preliminary data were collected by researchers and took approximately 10 minutes.

Introductory Information Form: This form developed by the researcher consists of 7 close-ended questions about demographic characteristics (age, sex, marital status, body mass index (BMI), feeding method used, etc.), medical records (chronic diseases, number of medications used), and level of mucositis.

### Constipation Severity Scale

Constipation severity scale (CSS) was developed by Varma et al^8^ in 2008. Scale’s validity and reliability in the Turkish language were tested by Kaya and Turan^9^, and it is a scale aiming to determine individuals’ frequency, intensity, and difficulty of defecation. Moreover, it was also aimed to measure the constipation symptoms via this scale. The scale consists of 16 items. Constipation severity scale has 3 sub-dimensions such as obstructive defecation, colonic inertia, and pain. The score to be obtained from “obstructive defecation” sub-dimension ranges between 0 and 28 points, the score to be obtained from “colonic inertia” sub-dimension between 0 and 29 points, and the score to be obtained from “pain” sub-dimension between 0 and 16 points. The minimum total score in CSS is 0 and the highest score is 73. The higher scores indicate more severe symptoms.^8,9^ Kaya and Turan^9^ calculated the Cronbach’s coefficient to vary between 0.92 and 0.93. In this study, Cronbach’s α coefficient was found to be 0.965.

### Mini Nutritional Assessment Test

Mini Nutritional Assessment Test recommended by “European Society of Parenteral and Enteral Nutrition” (ESPEN) was used for assessing the nutritional statuses of elders aged 65 years or more in nursing homes and hospitals. This test is a rapid method taking approximately 10-15 minutes. Developed by Vellas et al^10^, this method is widely used throughout the world. Mini Nutritional Assessment Test has 2 sub-dimensions as scanning and assessment and consists of 18 items in total. In this study, Cronbach’s *α* coefficient was found to be 0.842.

### Mucositis Assessment Indices

“Patient complaints index (PCI)” and “researcher mucositis index (RMI)” were originally developed in 1991 in the USA by Mahood et al^11^ based on the World Health Organization’s Mucositis Assessment System. In Turkey, it was used in 1995 by Sener^12^ in investigating the role of oral hygiene for patients receiving chemotherapy and the role of the use of chlorhexidine mouthwash (3×1) in incidence and severity of mucositis. Patient complaints index, RMI, and PCI, which are actually the same, are called RMI when examining the patients.

### Researcher Mucositis Index

Researcher mucositis index was filled in by the researcher based on the physical examination findings on the 1st and 15th days of observation and scored between 0 and 4 depending on the severity of oral mucositis—“0” refers to no oral mucositis, “1” refers to pain-free and minimal erythema or ulcers in mouth, “2” refers to painful erythema, edema, or ulcers in mouth and patient being capable of eating solid foods, “3” refers to painful erythema, edema, or ulcers in mouth and patient being incapable of eating solid foods, and “4” refers to need for parenteral or EN (through nasogastric tube).^11,12^

### Visual Analog Scale

It is a simple, effective, and reproducible pain measurement method that requires minimal instrument. Visual analog scale is widely used in clinical and laboratory cases, in which it is desired to rapidly measure the severity of pain. Points are marked on a 10 cm line, one end of which indicates very good (0 = no pain severity), and the other end indicates very bad (10 = most severe). The distance between the mark and the lowest end of line (0 = no pain) is measured, and the result indicates the numerical value of the patient’s pain severity.^13^

### Gastrointestinal Symptom Grading Scale

Gastrointestinal symptom grading scale (GSGS) was developed by Revicki et al^14^ in order to assess the symptoms frequently seen in GIS disorders. Validity and reliability of GSGS in the Turkish language were tested by Turan et al.^15^ Gastrointestinal symptom grading scale aims to determine how an individual felt because of gastrointestinal problems in the last week. Gastrointestinal symptom grading scale consists of 15 items and has 5 sub-dimensions as stomach ache, reflux, diarrhea, indigestion, and constipation. In this scale, first, fourth, and fifth items are about stomach ache, second and third items are about reflux, 11th, 12th, and 14th items are about diarrhea, sixth, seventh, eighth, and ninth items are about indigestion, and 10th, 13th, and 15th items are about constipation. Each item is rated between “No complaint” and “Very severe complaint” by using a 5-point Likert scale. The higher scores indicate more severe symptoms.^15^ The scale’s Cronbach’s *α* coefficient was reported to vary between 0.92 and 0.93. In this study, Cronbach’s *α* coefficient was found to be 0.965.

### Ethical Considerations

This study was carried out in accordance with the principles of the Helsinki Declaration. Before the study, ethics committee approval was obtained from Osmaniye Korkut Ata University’s Scientific Research and Publication Ethics Unit (date of 12.05.2018 and decree number of 2018/16/2). Anonymity and privacy were ensured throughout the study.

### Statistical Analysis

Statistical Package for Social Sciences (SPSS) statistical software for Windows Version 21.0 (IBM Corp.; Armonk, NY, USA) package program was used for data evaluation and statistical analysis. Introductory characteristics of the participants are expressed as mean values, SD, and percentages.

In examining the relationships between 2 qualitative variables, “continuity correction” or “χ^2^-cross tables” were used depending on the expected value levels. The fit of study data to normal distribution was tested using Kolmogorov–Smirnov and Shapiro–Wilk tests.

When comparing the results of 2 independent groups for the nonnormally distributed data, “Mann–Whitney *U*” test (*Z*-table values) statistics were used. When examining the relationship between 2 quantitative variables, Pearson’s correlation coefficient was used for those having normal distribution and Spearman’s correlation coefficient for those not having normal distribution. Statistical significance was set at *P* < .05 in comparisons.

## RESULTS

Table 1 shows the nutritional routes of patients treated in intensive care and sociodemographic characteristics of enterally and parenterally nutritional patients.

Nutritional status was found to have no statistically significant relationship with age group, sex, marital status, intensive care unit, BMI, and mucositis classes (*P* > .05). Comparing the scores of individuals receiving EN and PN as seen in Table 2, there was a statistically significant difference between mean MNA scores (Z = –6,611; *P* = .000). Total MNA scores of those receiving EN were significantly higher than the scores of those having PN. It was found that those receiving PN had malnutrition and those receiving EN were at risk.

A statistically significant difference was found between those receiving EN support and PN support in terms of MNA scanning scores (Z = –6.173; *P* = .000). Mini Nutritional Assessment Test scanning scores of those receiving EN were found to be statistically significantly higher than those of participants receiving PN support (Table 2). Considering the nutrition status, there was a statistically significant difference between MNA assessment scores (Z = –5.964; *P* = .000). Mini Nutritional Assessment Test assessment scores of those receiving EN were statistically significantly higher than those of individuals receiving PN (Table 2). A statistically significant difference was found between the groups in terms of the total gastrointestinal symptom scores (Z = –4.910; *P* = .000). Total GIS scores of those receiving PN were statistically significantly higher than those of individuals receiving EN. It was found that the severity of GIS was higher among those receiving PN when compared to those receiving EN (Table 2).

Comparing the scale scores of those receiving EN and PN supports presented in Table 3, it was determined that among those receiving EN, there was a negative relationship between mucositis index and colonic inertia score (*r* = –0.277; *P* = .041); positive relationship between MNA total scores and MNA scanning (*r* = 0.832; *P* = 0.000) and MNA assessment scores (*r* = 0.871; *P* = 0.000) and CSS’s pain sub-dimension (*r* = 0.321; *P* = .017) (*P* < .05). Among those receiving EN, there were statistically significant positive relationships between MNA scanning and MNA assessment scores (*r* = 0.497; *P* = .000) (*P* < .05). Among those receiving EN, there was a statistically significant positive relationship between CSS total scores and gastrointestinal symptom scale (*r* = 0.343; *P* = .010) and a CSS’s pain sub-dimension scores (*r* = 0.739;* P* = .000) and colonic inertia (*r* = 0.658; *P* = .000), and obstructive defecation (*r* = 0.737; *P* = .000) (*P* < .05).

According to the findings we obtained, a positive, high degree, and statistically significant relationship was found between MNA total scores and MNA scanning (*r* = 0.794; *P* = .000). and MNA assessment scores in EN (*r* = 0.751; *P* = .000) (*P* < .05). It was determined that positive weak and statistically significant relationship was found between MNA screening scores and MNA assessment scores in enteral feds (*r* = 0.497; *P* = .000). According to our findings, there was a weak positive and statistically significant relationship between MNA scanning scores (*r* = 0.321; *P* = .017) and the pain sub-dimension of the CSS in EN (*r* = 0.316; *P* = .019). According to the findings we obtained, statistically significant relationship was found between pain sub-dimension of the CSS and CSS (*r* = 0.739; *P* = .000), obstructive defecation (*r* = 0.394; *P* = .003), colonic inertia (*r* = 0.407; *P* = .002), and GIS total score (*r* = 0.474; *P* = .000).

As presented in Table 3, there was a statistically significant positive relationship between MNA total scores and MNA scanning (*r* = 0.794; *P* = .000) and MNA assessment scores (*r* = 0.751; *P* = .000) a statistically significant positive relationship between MNA total scores (*r* = 0.794; *P* = .000) and negative relationship GIS scale score (*r* = –0.395; *p* = .005) (*P* < .05). Among those receiving PN, there was a statistically significant and weak negative relationship between MNA scanning scores and GIS scales (*r* = –0.492; *P* = .005). Among those receiving PN, there was a statistically significant positive relationship between CSS total scores and scores in obstructive defecation (*r* = 0.800; *P* = .000) colonic inertia (*r* = 0.897; *P* = .000) and pain sub-dimensions of CSS (*r* = 0.858; *P* = .000) (*P* < .05). A statistically significant positive relationship was found between CSS’s pain sub-dimension scores and obstructive defecation (*r* = 0.518; *P* = .000) and colonic inertia sub-dimension scores (*r* = 0.804; *P* = .000) (*P* < .05). Among the ones receiving PN, a statistically significant positive relationship was found between obstructive defecation and colonic inertia sub-dimension scores of CSS (*r* = 0.583; *P* = .000).

As seen in Table 4, it was determined that the mean score in oral mucositis measurements for all the intensive care patients was 3.05 ± 0.79. The oral mucositis cases of the intensive care patients were found to be painful erythema, edema, or ulcer. In parallel with the results achieved here, it was found that the constipation score of intensive care patients was 44.23 ± 8.53, which indicates moderate level of constipation. Given the results achieved in this study, the malnutrition status of patients receiving enteral or PN support was found to be 6.50 ± 3.55. Malnutrition risk was detected in patients receiving treatment. The mean CSS of intensive care patients receiving enteral or parental nutrition was found to be 44.23 ± 8.53. It was found that these patients receiving intensive care treatment had moderate level of constipation severity and the mean score of intensive care patients in GIS symptom severity was found to be 67.02 ± 20.73, which indicates a high GIS symptom severity level.

## DISCUSSION

Examining the sociodemographic characteristics of patients receiving enteral or PN support in intensive care, it was determined that the patients receiving treatment were older than 65 years. In their study, Sachdev et al^16^ determined that the need for EN is higher among patients aged 60 years or older when compared to younger adults. In literature, it was reported that those needing nasogastric tube most were elderly adult individuals receiving treatment in a hospital.^16,17^ In this study, the GIS symptoms of patients receiving enteral and PN support were examined. Given the results obtained from this study, it can be stated that, among individuals having EN, increases were observed in constipation and its components together with increasing malnutrition, colonic inertia, and GISs. In the literature, it has been determined that the incidence of gastrointestinal complications is high in EN Moreover, it was also reported that the most common GIS symptom observed among patients receiving EN was constipation.^18^ In literature, it was reported that among the intensive care patients, constipation developed in relation with EN.^17,18^ It has been reported that constipation is caused by insufficient fluid intake, low residual solution use, and decreased intestinal motility. In the study of Kadamani et al^19^, it was determined that the incidence of constipation in patients fed continuously enterally was higher than in patients fed as bolus. In the study, it was determined that more than half of the intensive care patients had constipation and diarrhea.^20^ In several studies on constipation in intensive care setting, it was reported that constipation is related with organ deficiency, prolonged hospitalization times, and EN, but there were also many factors that may cause constipation.^21,22^ According to these findings, it was determined that together with increasing score of CSS, the patients having EN had higher scores in GIS symptom scale. The frequency of complications related to EN indicates the quality of care given to patients; therefore, their frequency needs to be determined.^17^ Identifying the most common complications related with EN in patients, determining the possible relationships between these problems and other aspects such as tube location, nutritional formulations, and methods of administration, and evaluating the quality of the care plan developed by the study unit for patients receiving EN are very important in preventing complications. It was also found that 65%-75.3% of intensive care patients receiving EN support had malnutrition.^5,6^ These findings are in corroboration with the results achieved in this study.^19^ Given the results obtained here, it can be stated that among the individuals having EN support, colonic inertia score increased with increasing oral mucositis. Since the malnutrition level is higher among the patients having limited oral feeding, development of oral mucositis would be inevitable. In a meta-analysis study, it was determined that although diarrhea ileus, anastomotic leak, and peritonitis are observed in intensive care patients having PN, it is a nutrition method that might cause more complication when compared to enteral method.^23^ Oral mucositis is a weakening condition that might harm the normal tissue and is caused by deep mucosal ulcers.^7^ Mucositis causes pain in the mouth and it negatively affects the patients’ quality of life by leading to decreased food intake and resulting in malnutrition.^8-10^ In a study carried out by Andersson et al^24^, it was found that oral health problems are more common among undernourished patients. In the earlier studies, it was revealed that the oral mucosal membrane of patients, who cannot feed orally, was in very bad condition. Other studies corroborate the results of that study.^12,24^ In another study, it was determined that Gram-negative bacteria were more common among patients receiving EN.^25^ On the other hand, the ones receiving PN were found to have more GIS together with increasing MNA total scores. Although PN is a life-saving treatment in patients, infections and complications related to technical complications may develop. Metabolic complications associated with PN in adult patients include hyperglycemia, hypoglycemia, hyperlipidemia, hypercapnia, acid–base disorders, and liver complications. The frequency and severity of these complications may also vary depending on the patient and PN-specific factors. Appropriate assessment of the patient’s nutritional status, determination of fluid and electrolyte requirements, adaptation to the patient’s chronic diseases, clinical condition and drug therapy, and monitoring the patient’s tolerance and response to nutritional support are very important to avoid these complications. Knowledge of treatments for early recognition and management of these complications is essential.^26^ In an earlier study, a comparison was made between patients receiving PEG and those receiving total PN (TPN) among the patients having treatment in intensive care unit, and it was determined that malnutrition status of those receiving PEG (enteral) nutrition was better than those receiving TPN (parenteral) nutrition. It was reported that it is necessary to provide the patients in intensive care unit the nutrition support as immediate as possible in order to reduce the morbidity, mortality, and malnutrition and PEG procedure is a safer and more effective nutrition support when compared to the other procedures.^18^ In the study of Nasiri et al^27^ in which they compared bolus and continuous enteral feeding, it was found that there was no statistically significant difference between constipation, diarrhea, vomiting, abdominal distension, and gastric residual volume. Although abdominal distention, diarrhea, and high gastric residual volume were observed in their study by Serpa et al^28^ more frequently, in intermittent feeding patients than in continuous enteral fed patients, no statistically significant difference was found between them. In the study of Lee et al^29^, pneumonia was more common in patients fed as bolus compared to those fed continuously enteral. It is thought that the variation in the complications associated with EN is due to the diversity of the EN protocols and nutritional solutions used by the institutions, the care methods applied by the nurses, and the individual differences of the patients. Therefore, it is very important to define the complications of the GIS that may develop due to EN.

There are several limitations of this study. This study is limited to the patients who were treated in intensive care units between January and June 2019 and participated in the study on enteral or PN. Therefore, the results can only be generalized to the patients included in this study. In addition, intensive care patients are patients with multiple health problems. The possibility of these problems leading to nutritional changes in patients other than physical fixation is another limitation of the study. The heterogeneity of medical diagnoses, treatments, and conscious states of the patients included in the study was considered as a limitation of the study. In addition, the short follow-up period of the study is among the limitations of the study. Despite these limitations and the limited number of cases, we strongly believe that our study will provide an important contribution to the studies in this field. Our study may help against uncertainties that may occur before patients begin EN or PN. Although more comprehensive studies are needed on this subject, we hope that the results of our study will shed light on the literature.

## Conclusion

Oral mucositis cases observed among intensive care patients receiving EN or PN support were found to be painful erythema, edema, or ulcers. It was found that constipation level of intensive care patients was moderate. Given the results achieved in this study, malnutrition risk was found in intensive care patients receiving EN or PN. It was determined that patients receiving EN or PN in intensive care unit had moderate level of constipation severity and high level of GIS symptom severity. These findings are very important since there are only few studies on the effects of PN and EN methods. Further studies should be carried out in larger centers with higher number of patients and on different sample groups.

## Figures and Tables

**Table 1.** Sociodemographic Characteristics of Patients Having Enteral and Parenteral Nutrition

Variable (*n* = 104)	Enteral (*n* = 55)		Parenteral (*n* = 49)		Total		Statistical Analysis^*^ Probability
	n	%	n	%	n	%	
*Age groups*							
18-45	3	5.5	1	2.0	4	3.8	χ^2^ = 1.191 *P* = .551
46-65	17	30.9	13	26.6	30	28.8	
>65	35	63.6	35	71.4	70	67.4	
*Sex*							
Female	34	61.8	24	49.0	58	55.8	χ^2^ = 1.250 *P* = .264
Male	21	38.2	25	51.0	46	44.2	
*Marital status*							
Married	21	38.2	22	44.9	43	41.3	χ^2^ = 0.245 *P* = .621
Single	34	61.8	27	55.1	61	58.7	
*Intensive care*							
Surgery	19	34.5	14	28.6	33	31.7	χ^2^ = 3.055 *P* = .217
Coronary	17	31.0	10	20.4	27	26.0	
Internal	19	34.5	25	51.0	44	42.3	
*BMI class*							
Lean	33	60.0	25	51.0	58	55.8	χ^2^ = 2.319 *P* = .509
Normal	12	21.8	17	34.7	29	27.9	
Overweight	8	14.6	5	10.2	13	12.5	
Obese	2	3.6	2	4.1	4	3.8	
*Mucositis class*							
Mild	1	1.8	1	2.0	2	1.9	χ^2^ = 6.147 *P* = .105
Moderate	18	32.7	6	12.3	24	23.1	
Severe	21	38.2	24	49.0	45	43.3	
Requiring parenteral/enteral support	15	27.3	18	36.7	33	31.7	

^*^In examining the relationships between 2 qualitative variables, “continuity correction” or “*χ*^2^-cross tables” were used depending on the expected value levels.

BMI, body mass index.

**Table 2.** Comparison of the Scale Scores Between the Groups

Variable (*n* = 104)	Enteral (*n* = 55)		Parenteral (*n* = 49)		Statistical Analysis^*^ Probability
	X±S.S.	Median (IQR)	X±S.S.	Median (IQR)	
Mucositis scale	2.91 ± 0.82	3.0 (2.0)	3.20 ± 0.73	3.0 (1.0)	*Z* = –1.922 *P* = .055
VAS	5.60 ± 1.40	5.0 (1.0)	6.00 ± 1.77	6.0 (3.0)	Z = –1.390 *P* = .165
MNA total score	17.73 ± 5.06	17.0 (7.5)	7.78 ± 4.03	7.0 (5.5)	*Z* = –6.611 ***P*** ** = .000**
MNA scanning	7.27 ± 2.51	7.0 (3.0)	3.47 ± 2.52	3.0 (3.0)	Z = –6.173 ***P*** ** = .000**
MNA assessment	8.45 ± 3.22	9.0 (4.0)	4.31 ± 2.47	4.0 (4.0)	*Z* = –5.964 ***P*** ** = .000**
CSS total score	43.67 ± 5.88	45.0 (7.0)	44.86 ± 10.79	45.0 (10.0)	*Z* = –0.777 *P* = .437
Obstructive defecation	18.56 ± 2.53	19.0 (3.0)	18.36 ± 3.74	19.0 (4.0)	*Z* = –0.453 *P* = .651
Colonic inertia	15.80 ± 2.26	16.0 (3.0)	16.53 ± 4.22	16.0 (5.5)	*Z* = –0.427 *P* = .670
Pain	9.31 ± 2.85	10.0 (3.0)	9.96 ± 3.99	10.0 (5.0)	*Z* = –1.176 *P* = .239
Total gastrointestinal symptom	56.43 ± 12.90	57.0 (12.0)	78.90 ± 21.51	90.0 (34.5)	*Z* = –4.910 ***P*** ** = .000**

^*^When comparing the results of 2 independent groups for the nonnormally distributed data, “Mann–Whitney *U*” test (*Z*-table values) statistics were used.

MNA, Mini Nutritional Assessment Test; VAS, visual analog scale.

**Table 3.** Relationship Between Scale Scores and Groups

Variable			Mucositis Index	VAS	MNA Total	MNA Scan	MNA Assessment	CSS Total	OD	CI	Pain	GIS
Enteral (n = 5 5)	Mucositis index	*r* *P*	1.000 .	0.200 .142	–0.060 .662	0.009 .946	–0.112 .415	–0.230 .092	–0.045 .745	–**0.277** **.041**	–0.198 .147	0.044 .752
	VAS	*r* *P*	0.200 .142	1.000 .	–0.057 .680	–0.145 .291	0.038 .783	0.000 .999	0.072 .601	–0.141 .306	–0.089 .518	–0.145 .291
	MNA total	*r* *P*	–0.060 .662	0.057 .680	1.000 .	**0.832** **.000**	**0.871** **.000**	0.052 .704	0.023 .866	0.073 .597	**0.321** **.017**	0.116 .398
	MNA scanning	*r* *P*	0.009 .946	–0.145 .291	**0.832** **.000**	1.000 .	**0.497** **.000**	0.102 .458	–0.016 .908	0.082 .553	**0.316** **.019**	0.138 .314
	MNA assessment	*r* *P*	–0.112 .415	0.038 .783	**0.871** **.000**	**0.497** **.000**	1.000 .	–0.015 .916	–0.011 .938	–0.021 .877	0.240 .077	0.127 .357
	CSS	*r* *P*	–0.230 .092	0.000 .999	0.052 .704	0.102 .458	–0.015 .916	1.000 .	**0.737** **.000**	**0.658** **.000**	**0.739** **.000**	**0.343** **.010**
	Obstructive defecation	*r* *P*	–0.045 .745	0.072 .601	0.023 .866	–0.016 .908	0.118 .392	–0.011 .938	1.000 .	0.117 .393	**0.394** **.003**	0.210 .124
	Colonic inertia	*r* *P*	–0.277 **.041**	–0.141 .306	0.073 .597	0.082 .553	–0.021 .877	**0.658** **.000**	0.117 .393	1.000 .	**0.407** **.002**	0.214 .116
	Pain	*r* *P*	–0.198 .147	–0.089 .518	**0.321** **.017**	**0.316** **.019**	0.240 .077	**0.739** **.000**	0.394 .03	**0.407** **.002**	1.000 .	**0.474** **.000**
	GIS total	*r* *P*	0.044 .752	–0.145 .291	0.116 .398	0.138 .314	0.127 .357	**0.343** **.010**	0.210 .124	0.214 .116	**0.474** **.000**	1.000 .
Parenteral (n = 49)	Mucositis index	*r* *P*	1.000 .	–0.128 .380	0.000 .998	–0.019 .897	0.015 .920	–0.001 .993	0.031 .833	0.068 .644	–0.104 .478	–0.263 .068
	VAS	*r* *P*	–0.128 .380	1.000 .	0.020 .891	–0.174 .232	0.182 .211	0.236 .103	0.096 .512	0.207 .154	0.188 .195	0.233 .106
	MNA total	*r* *P*	0.000 .998	0.020 .891	1.000 .	**0.794** **.000**	**0.751** **.000**	–0.032 .827	0.015 .919	–0.114 .437	–0.164 .261	–**0.395** **.005**
	MNA scanning	*r* *P*	–0.019 .897	–0.174 .232	**0.794** **.000**	1.000 .	0.241 .095	–0.192 .186	–0.134 .360	–0.254 .078	–0.215 .138	–**0.492** **.000**
	MNA assessment	*r* *P*	0.015 .920	0.182 .211	**0.751** **.000**	0.241 .095	1.000 .	0.210 .148	0.257 .075	0.090 .539	0.033 .820	–0.084 .567
	CSS	*r* *P*	–0.001 .993	0.236 .103	–0.032 .827	–0.192 .186	0.210 .148	1.000 .	**0.800** **.000**	**0.897** **.000**	**0.858** **.000**	0.159 .274
	Obstructive defecation	*r* *P*	0.031 .833	0.096 .512	0.015 .919	–0.134 .360	0.257 .075	**0.800** **.000**	1.000 .	**0.583** **.000**	**0.518** **.000**	0.113 .440
	Colonic inertia	*r* *P*	0.068 .644	0.207 .154	–0.114 .437	–0.254 .078	0.090 .539	**0.897** **.000**	**0.583** **.000**	1.000 .	**0.804** **.000**	0.070 .633
	Pain	*r* *P*	–0.104 .478	0.188 .195	–0.164 .261	–0.215 .138	0.033 .820	**0.858** **.000**	**0.518** **.000**	**0.804** **.000**	1.000 .	0.185 .204
	GIS total	*r* *P*	–0.263 .068	0.233 .106	–0.395 .005	–0.492 .000	–0.084 .567	0.159 .274	0.113 .440	0.070 .633	0.185 .204	1.000 .

^*^When examining the relationship between 2 quantitative variables, Pearson’s correlation coefficient was used for those having normal distribution and Spearman’s correlation coefficient for those not having normal distribution.

CSS, constipation severity scale; GIS, gastrointestinal system; MNA, mini nutritional assessment test; OD, obstructive defecation; VAS, visual analog scale.

**Table 4.** Distribution of Scale Scores of Patients Receiving Enteral and Parenteral Nutrition

Scales	Mean	SD	Median	Minimum	Maximum
Mucositis index	3.05	0.79	3.0	1.0	4.0
VAS	5.79	1.58	6.0	2.0	10.0
MNA total score	11.98	6.07	12.0	1.0	23.5
MNA scanning	5.48	3.15	6.0	0.0	13.0
MNA assessment	6.50	3.55	7.0	0.0	12.5
Constipation severity total	44.23	8.53	45.0	8.0	65.0
Obstructive defecation	18.47	3.15	19.0	4.0	26.0
Colonic inertia	16.14	3.33	16.0	4.0	26.0
Pain	9.62	3.43	10.0	0.0	16.0
Gastrointestinal symptom	67.02	20.73	60.0	34.0	99.0

MNA, mini nutritional assessment test; VAS, visual analog scale.
